# Achievement motivation and mental health among medical postgraduates: the chain mediating effect of self-esteem and perceived stress

**DOI:** 10.3389/fpsyg.2024.1483090

**Published:** 2024-10-23

**Authors:** Mu-yun Ma, Yao Li, Li Guo, Guan-e Yang

**Affiliations:** ^1^Department of Applied Psychology, School of Humanities and Social Sciences, Shanxi Medical University, Jinzhong, China; ^2^Department of Traditional Chinese Pharmacology, School of Pharmaceutical Sciences, Shanxi Medical University, Jinzhong, China

**Keywords:** medical postgraduates, achievement motivation, depression, anxiety, self-esteem, perceived stress

## Abstract

**Introduction:**

Medical postgraduates generally experience high levels of depression and anxiety. Previous studies have investigated the impact of various achievement motivations on depression/anxiety among medical students.

**Methods:**

This study focused on self-esteem and perceived stress, examining the internal mechanisms through which achievement motivation affects depression/anxiety. 530 medical postgraduate students (66.04% female and 33.96% male) were administered the Achievement Goal Orientation Scale, Self-Esteem Scale, Perceived Stress Scale, and Depression-Anxiety-Stress Scale.

**Results:**

Results indicated that: (1) mastery-approach goals were negatively correlated with depression/anxiety; mastery-avoidance goals were positively correlated with depression/anxiety; performance-avoidance goals positively predicted depression/anxiety; (2) self-esteem mediated the relationship between achievement motivation and depression/anxiety; (3) perceived stress played a mediating role in the relationship between achievement motivation and depression/anxiety; (4) self-esteem and perceived stress played a chain mediating role in the relationship between achievement motivation and depression/anxiety; (5) there was no significant linear correlation between performance-approach goals and depression/anxiety.

**Discussion:**

Although this study employed a cross-sectional design and self-report scales, both of which have certain limitations, the findings still hold significant theoretical and practical implications. The research reveals a mediating pathway between achievement goals and mental health, offering new insights into mental health education for medical graduate students.

## Introduction

Depression and anxiety are key indicators in assessing individual mental health. Depression is associated with low mood, reduced learning or work efficiency, insomnia, and an increased risk of suicide (Liu and Bai, 2014). Anxiety is related to lower subjective well-being, poorer sleep quality, and increased procrastination (Huang et al., 2023; Malone and Wachholtz, 2018; Zhou A.-x., 2022). Notably, mental health problems among medical postgraduates are prominent (Dong et al., 2020; Tan et al., 2021; Zhou et al., 2022), with detection rates of depression and anxiety at 37.1 and 26.8% (Zhao et al., 2019). As the backbone of China’s future healthcare industry, the psychological health of medical students is crucial in reducing their attrition and preventing the loss of medical talent (Zhou W., 2022), which is of great significance to China’s healthcare industry (Mao et al., 2020). This study aims to examine the relevant factors affecting the mental health of medical postgraduates and provide recommendations for cultivating qualified highly educated medical talents.

Achievement motivation is an important psychological driving force that affects an individual’s level of effort when pursuing success (Yang et al., 2016). Varied achievement motivations can lead to different mental health conditions (Hu and Lin, 2010; Kuroda and Sakurai, 2011; Vergara and Roberts, 2011). Achievement motivation was initially divided into two dimensions: approach goals and avoidance goals. Subsequently, Elliot (1999) and Pintrich (2000) proposed achievement goals including mastery-approach goal (MAP), mastery-avoidance goal (MAV), performance-approach goal (PAP), performance-avoidance goal (PAV). When individuals hold a MAP orientation, they strive to master learning and work tasks to enhance their abilities. Individuals who hold an MAV orientation are more concerned about avoiding misunderstandings of knowledge. Individuals holding a PAP orientation focus on showcasing their abilities and expect to receive positive feedback from others. Individuals who hold a PAV orientation are afraid of exposing their flaws and receiving negative evaluations. Previous longitudinal studies have shown that achievement motivation is a dynamic process. There have been many longitudinal and cross-sectional studies summarizing the relationship between achievement motivation and mental health. Some longitudinal studies with Chinese college students as participants have found that achievement goals among college students decrease from freshman to junior year, but rebound in senior year (Liu et al., 2023; Liu et al., 2024). The anxiety problem of college students is alleviated during their 4 years in college. In the longitudinal trajectories of achievement goals, except for the performance-avoidance goal, more students belong to the decreasing class than to the increasing class. These data indicate that achievement motivation and mental health are dynamic processes. Previous studies have shown that MAP negatively predicts depression and anxiety (Liu and Guo, 2003; Wang et al., 2021; Zhang, 2019), indicating that a MAP may offer a protective effect on depression and anxiety. A PAV positively predicts depression and anxiety (Wang et al., 2022; Wang et al., 2021). Individuals who hold PAV are concerned about receiving negative feedback from others while achieving their goals, and therefore experience more depression and anxiety. A MAV is significantly associated with increased anxiety, negative emotions, and fear of failure (Sideridis, 2008), especially in stressful situations where individuals tend to develop a fear of failure. The relationship between PAP and emotions is relatively complex. Some studies have shown that a PAP orientation is negatively correlated with depression and anxiety (Sideridis, 2005). However, other studies have reached opposite conclusions (Linnenbrink and Pintrich, 2000). Therefore, we assume that:

*Hypothesis 1a*: A MAP may relieve symptoms of depression and anxiety.

*Hypothesis 1b*: A MAV may make individuals more vulnerable to depression and anxiety.

*Hypothesis 1c*: A PAV is a risk factor for mental symptoms.

However, existing studies have primarily explored the direct relationship between different dimensions of achievement motivation and depression/anxiety, yet the internal mechanisms remain unclear. Ames points out that achievement goals are a flexible motivational structure that is influenced by situational factors. Mischel (1990) emphasizes that when exploring achievement motivation, it is necessary to consider individual personality traits, as well as value situational factors. Therefore, current research introduces self-esteem and perceived stress to analyze the impact mechanism of achievement motivation on depression/anxiety among medical students.

Self-esteem is a positive or negative attitude toward themselves (Rosenberg, 1965). The Terror Management Theory (TMT) posits that self-esteem is an experience of one’s value and sense of meaning, which can alleviate anxiety caused by inherent fears of death (e.g., failure, forgetting, and rejection) (Greenberg et al., 1986), symbolically transcending death and promoting mental health. Therefore, self-esteem serves as a psychological mechanism for adapting to the environment and reducing anxiety. The Vulnerability Model indicates that low self-esteem is one reason individuals experience depressive symptoms (Gao et al., 2015). The negative self-schema of individuals constitutes cognitive susceptibility to depression. Therefore, low self-esteem is related to psychological problems (Huang, 2021; Ostrov, 1982; Sahlan et al., 2021). Self-esteem can maintain mental health (Macinnes, 2006; Rosenberg, 1979) and buffer the impact of negative events (Gerard and Buehler, 2004; Moksnes et al., 2022). Additionally, self-esteem can be influenced by achievement motivation. Covington’s self-worth theory suggests that individuals are born with a need to maintain self-esteem by taking various measures, such as pursuing success to demonstrate their abilities or avoiding failure (Covington, 1998). Dweck believes that individuals with malleable intelligence theory tend to adopt mastery goals, while individuals with fixed intelligence theory tend to form performance goals and the helpless pattern. Once individuals perform worse or fall behind others, they may feel that their abilities are insufficient and worthless, resulting in low self-esteem. Research has supported this (Chen et al., 2017; Gębka, 2014; Heimpel et al., 2006; Komarraju and Dial, 2014; Shim et al., 2012), indicating that self-esteem is positively correlated with MAP and negatively correlated with MAV and PAV. However, the relationship between self-esteem and PAP remains inconsistent. Based on the above theoretical analysis and empirical research, we speculate that individuals who hold MAP have higher self-esteem and lower depression and anxiety. Individuals holding MAV and PAV are the opposite. Therefore, we hypothesize that:

*Hypothesis 2a*: Self-esteem meditates the association between MAP and depression/anxiety.

*Hypothesis 2b*: Self-esteem plays a mediating role between MAV and depression/anxiety.

*Hypothesis 2c*: PAV could further influence depression/anxiety through the meditation of self-esteem.

The Diathesis-Stress theory posits that mental health depends on the interplay between an individual’s inherent diathesis and external stressors (Liang, 2012). This suggests that symptoms of anxiety and depression are also influenced by perceived stress (Sun et al., 2022). Stress is a primary factor contributing to emotional disorders in individuals (Abela et al., 2007; Ren et al., 2011). In the collaborative training model of medical education, Chinese medical postgraduates face enormous academic and scientific research pressures, as well as clinical responsibilities such as documenting case histories and doctor-patient communication. Consequently, medical students are susceptible to various psychological problems (Wu et al., 2020). Nonetheless, not all medical students experience emotional imbalance under immense pressure. Stress affects individuals’ mental health through their characteristics such as motivation and cognition (Baumann et al., 2005; Mansell et al., 2023). Individuals with diverse motivational goals exhibit varied cognitive, emotional, and behavioral responses to stress. Dweck notes that performance goals are associated with a helpless mode and low self-esteem. Therefore, individuals with performance goals adopt a more conservative self-protection attitude and exhibit boredom while performing tasks. Those with master goals frequently adjust efforts and strategies in response to failure, attributing setbacks to strategy rather than ability (Diener and Dweck, 1978). Empirical research also demonstrates that perceived stress correlates not only with depression but also with goal orientation. In stressful situations, individuals pursuing performance goals are reluctant to make efforts, fearing that others attribute their (possible) failures to inadequate abilities (Vandewalle et al., 2019). Those with PAV tend to avoid failure and care about their performance in stressful situations, resulting in elevated anxiety and self-regulation failure (Sideridis, 2018). Further research reveals that, MAP has a protective effect on depression through the mediating effect of perceived stress, whereas PAV and perceived stress deepen depressive symptoms (Wang et al., 2021). Therefore, we hypothesize that:

*Hypothesis 3a*: MAP can reduce depression/anxiety through perceived stress.

*Hypothesis 3b*: Perceived stress could mediate the relationship between MAV and depression/anxiety.

*Hypothesis 3c*: PAV may aggravate symptoms via perceived stress.

**Figure 1:**
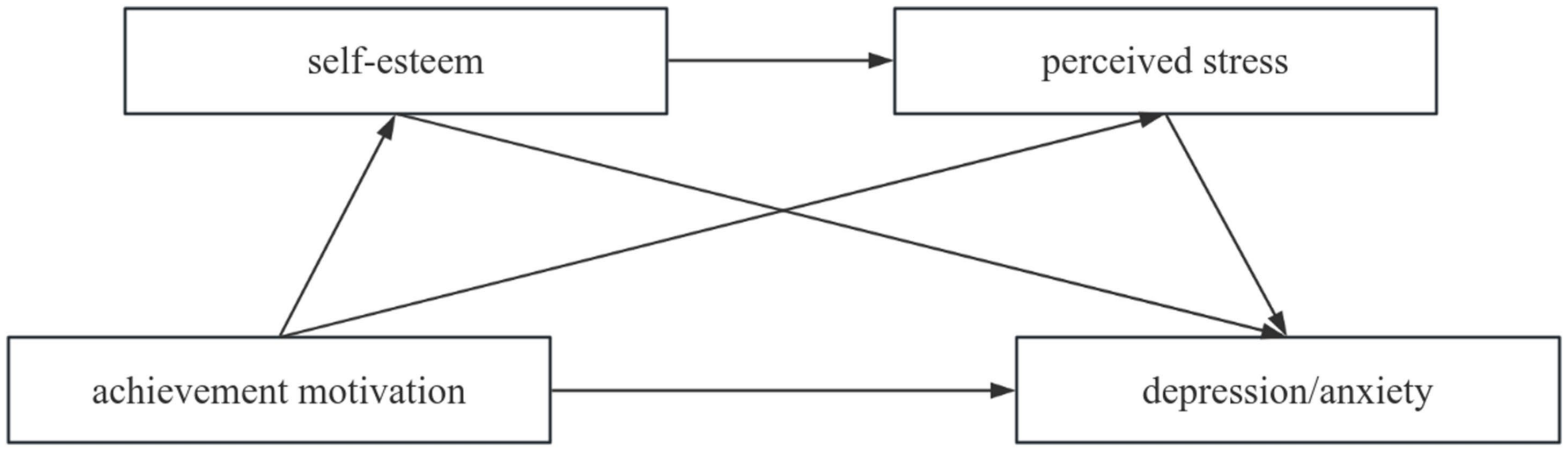
Conceptual model.

In addition, perceived stress is influenced by self-esteem. As an adaptive mechanism, individuals with high self-esteem have stronger adaptability and believe they can cope with challenges (Campbell and Foster, 2013). Self-esteem can alleviate the negative impact of stress on individuals and protect them (Byrne et al., 2007), enabling them to feel less stress (Zhu Y.-l. et al., 2023). Medical students with high self-esteem have a more positive and stable self-awareness, allowing them to adjust their mindset and adopt more positive coping strategies when faced with external pressure (Xu et al., 2022). However, postgraduates with low self-esteem tend to engage in negative self-attribution when struggling to withstand external pressure, leading to unreasonable self-evaluation and increased stress. Based on this, we propose that (Figure 1):

*Hypothesis 4a*: MAP is negatively related to depression/anxiety through the integrated mediation of self-esteem and perceived stress.

*Hypothesis 4b*: MAV could affect depression/anxiety through the serial mediation of self-esteem and perceived stress.

*Hypothesis 4c*: Self-esteem and perceived stress could play a chain-mediating role in the association between PAV and depression/anxiety.

## Methods

### Procedures

The protocol of the current study was approved by the Ethics Review Committee of the author’s organization. From August to December 2023, Data were collected at a medical university in Shanxi Province. Before data collection, Informed consent was obtained from all participants. Completion of the questionnaire did not affect course grades. The subjects filled out demographic information (such as age, gender, etc.), and completed questionnaires related to this study. If a participant requested an explanation of the questionnaire results, researchers would send the results to the participant’s email within a week.

### Participants

We adopted a convenient sampling method in this study. Medical postgraduate students were selected as participants in this survey. A total of 543 questionnaires were distributed, and 13 invalid questionnaires were excluded. We eliminated invalid questionnaires by specifying criteria such as instructed items, questionnaire completion time, continuity options, and outlier analysis to ensure questionnaire quality. First, we set up one “Instructed items” (“Please choose ‘strongly disagree’ for this question”), and the data of participants who did not choose this option were excluded. Secondly, due to the fact that the response time of participants to survey items is unlikely to exceed 2 s per item (Huang et al., 2012), and since this survey contained a total of 75 items, data from participants who completed the questionnaire for less than 150 s were excluded. Thirdly, if the number of consecutive responses exceeds half of the questionnaire length, we will consider it as invalid data. Additionally, researchers have paid attention to outliers that appeared in the participant’s answer data. For example, if a participant fills in an age below 20 years old, the data would be excluded. The research sample mainly came from China’s Shanxi Province, and may not represent all Chinese medical postgraduate students.

Finally, 530 medical students completed the questionnaires, yielding a response rate of 97.6%. All of the subjects were aged between 21 and 36 years old (*M* = 23.62, SD = 1.99). Of the participants, 350 were female (66.04%), 180 were male (33.96%); 257 came from rural areas (48.49%), 273 came from urban areas (51.51%);133 were single-child (25.09%), 397 were non-single-child (74.91%).

### Measures

#### General information

The general information of the questionnaire included age, gender, major, permanent residence, and whether the single child.

#### Achievement goal orientations scale

The Chinese revised version of the achievement goal orientations scale (Liu and Guo, 2003) was used to measure the achievement goals of medical postgraduate students, including 29 items. Each item was rated on a scale from 1 (never) to 5 (always). The questionnaire comprises four dimensions: mastery-approach goals, performance-approach goals, mastery-avoidance goals, and performance-avoidance goals. In our study, Cronbach’s *α* for each subscale were 0.856, 0.889, 0.814, and 0.866, respectively.

#### Self-esteem scale

We used the 10-item Chinese revision of the Rosenberg Self-Esteem Scale (Shen and Cai, 2008) to assess the degree of self-esteem. Subjects responded on a 4-point scale with values ranging from 1 (strongly disagree) to 4 (strongly agree). Higher scores indicated higher self-esteem. The Cronbach’s *α* of the scale in this study was 0.852.

#### Perceived stress scale

We adopted the Chinese version of the Perceived Stress Scale (CPSS) developed by Cohen and revised by Yang to measure perceived stress of participants (Cohen et al., 1983; Yang and Huang, 2003), which consists of 14 items. Given a 5-point scale, response options ranged from 0 (never) to 4 (most days). The scale is divided into two dimensions: nervousness and a sense of loss of control. In the study, Cronbach’s *α* for each subscale were 0.789 and 0.904.

#### Depression-anxiety-stress scale

This study used the Simplified Chinese version of the Depression Anxiety Stress Scale (DASS-21) compiled by Lovibond and Lovibond (1995) and revised by Gong et al. (2010), in which the Depression and Anxiety subscales measured depression and anxiety. Each of these two subscales comprises 7 items. Responses used the original 4-point format from 0 (strongly does not fit me) to 3 (strongly fits me). Higher scores on the subscale indicate more intense corresponding negative emotional experiences.

### Statistical analysis

We used SPSS 23.0 and PROCESS 4.0 plugins for data processing. Firstly, descriptive statistics and Pearson correlation analyses were initially conducted with SPSS 23.0. Secondly, the Common Method Bias test was performed using Harman’s single-factor test. Finally, integrated mediation analysis was conducted using Model 6 of the PROCESS macro program, employing the Bootstrap method with a sample size of 5,000. The significance of the integrated mediation effect was assessed within a 95% confidence interval.

## Results

### Common method bias test

Due to the use of self-report scales in our study, there may be common method biases. Harman’s single-factor test was employed to assess common method bias (Zhou and Long, 2004), revealing 11 factors with eigenvalues greater than 1 that were not rotated. The first factor accounted for 22.14% of the variance (<40%), so there was no severe common method bias in this study.

The descriptive statistics and correlation coefficients between variables are shown in Table 1. Mastery-approach goal (MAP) was negatively correlated with perceived stress (PS), depression (DS), and anxiety (AS), and positively correlated with self-esteem (SE). Mastery-avoidance goal (MAV) was positively correlated with PS, DS, and AS, and negatively correlated with SE. Performance-avoidance goal (PAV) was positively correlated with PS, DS, and AS, and negatively correlated with SE. There was no significant correlation between performance-approach goal (PAP) and PS, SE, DS, and AS.

**Table 1:** Descriptive statistics and correlations between variables.

	M	SD	1	2	3	4	5	6	7	8
1. MAP	29.86	5.81	1							
2. PAP	27.92	6.95	0.45***	1						
3. MAV	15.32	3.91	0.18***	0.44***	1					
4. PAV	13.24	4.90	−0.05	0.39***	0.56***	1				
5. SE	29.45	4.65	0.28***	0.03	−0.16***	−0.39***	1			
6. PS	23.17	6.93	−0.43***	0.01	0.35***	0.44***	−0.50***	1		
7. DS	4.40	6.64	−0.22***	0.04	0.20***	0.39***	−0.50***	0.50***	1	
8. AS	4.65	6.18	−0.18***	0.08	0.26***	0.41***	0.43***	0.51***-	0.83***	1

MAP, mastery-approach goal; PAP, performance-approach goal; MAV, mastery-avoidance goal; PAV, performance-avoidance goal; SE, self-esteem; PS, perceived stress; DS, depression; AS, anxiety. *N* = 530, *p < 0.05, **p < 0.01, ***p < 0.001.

### Test of chain meditation

In the study, Model 6 from the PROCESS plugin was used to analyze the chain meditation role of self-esteem (SE) and perceived stress (PS) in the relationship between achievement goals and depression (DS)/anxiety (AS).

### Analysis of the effect of mastery-approach goal (MAP) on depression/anxiety

Table 2, MAP positively predicted SE (*β* = 0.28, *p* < 0.001), and negatively predicted PS (*β* = −0.32, *p* < 0.001). SE significantly negatively predicted PS (*β* = −0.41, *p* < 0.001), DS (*β* = −0.33, *p* < 0.001), and AS (*β* = −0.24, *p* < 0.001). PS positively predicted DS (*β* = 0.35, *p* < 0.001) and AS (*β* = 0.42, *p* < 0.001). Self-esteem and perceived stress mediated the relationship between mastery-approach goal and depression/anxiety.

**Table 2:** The direct and indirect effects of MAP on depression and anxiety.

	Model 1			Model 2			Model 3		
	Outcome: SE			Outcome: PS			Outcome: DS (AS)		
	SE	β	t	SE	β	t	SE	β	t
MAP	0.03	0.28	6.76^***^	0.04	−0.32	−8.53^***^	0.05 (0.04)	0.02 (0.07)	0.56 (1.66)
SE				0.06	−0.41	−10.98^***^	0.06 (0.06)	−0.33 (−0.24)	−8.13^***^ (−5.65^***^)
PS							0.04 (0.04)	0.35 (0.42)	7.95^***^ (9.49^***^)
*R* ^2^	0.08			0.34			0.34 (0.31)		
*F*	45.68^***^			133.70^***^			88.61^***^ (77.26^***^)		

MAP, mastery-approach goal orientation; SE, self-esteem; PS, perceived stress; DS, depression; AS, anxiety. *p < 0.05, **p < 0.01, ***p < 0.001.

**Figure 2:**
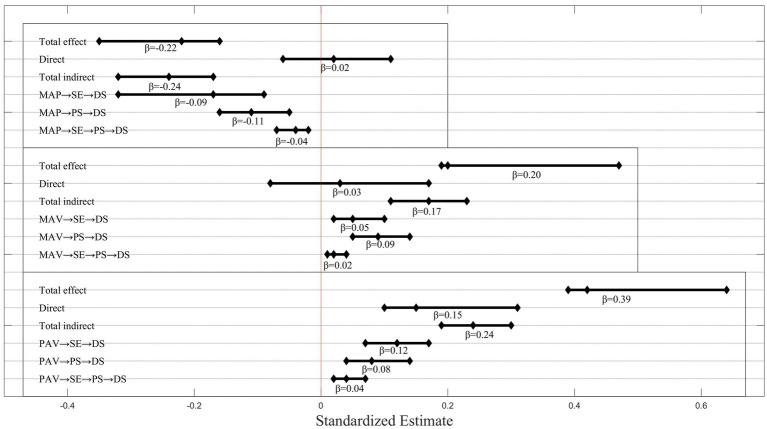
Effects from MAP, MAV, or PAV to DS.

Figure 2, the overall effect of MAP on DS was significant (*β* = −0.22, 95% CI = [−0.35, −0.16]), a non-significant direct effect (*β* = 0.02, 95% CI = [−0.06, 0.11]), and a significant indirect effect (*β* = −0.24, 95% CI = [−0.32, −0.17]). The indirect pathway from MAP to DS was mediated by SE (*β* = −0.09, 95% CI = [−0.32, −0.17]), PS (*β* = −0.11, 95% CI = [−0.16, −0.05]), and SE → PS (*β* = −0.04, 95% CI = [−0.07, −0.02]). Self-esteem and perceived stress played a complete mediating role between mastery-approach goal and depression.

**Figure 3:**
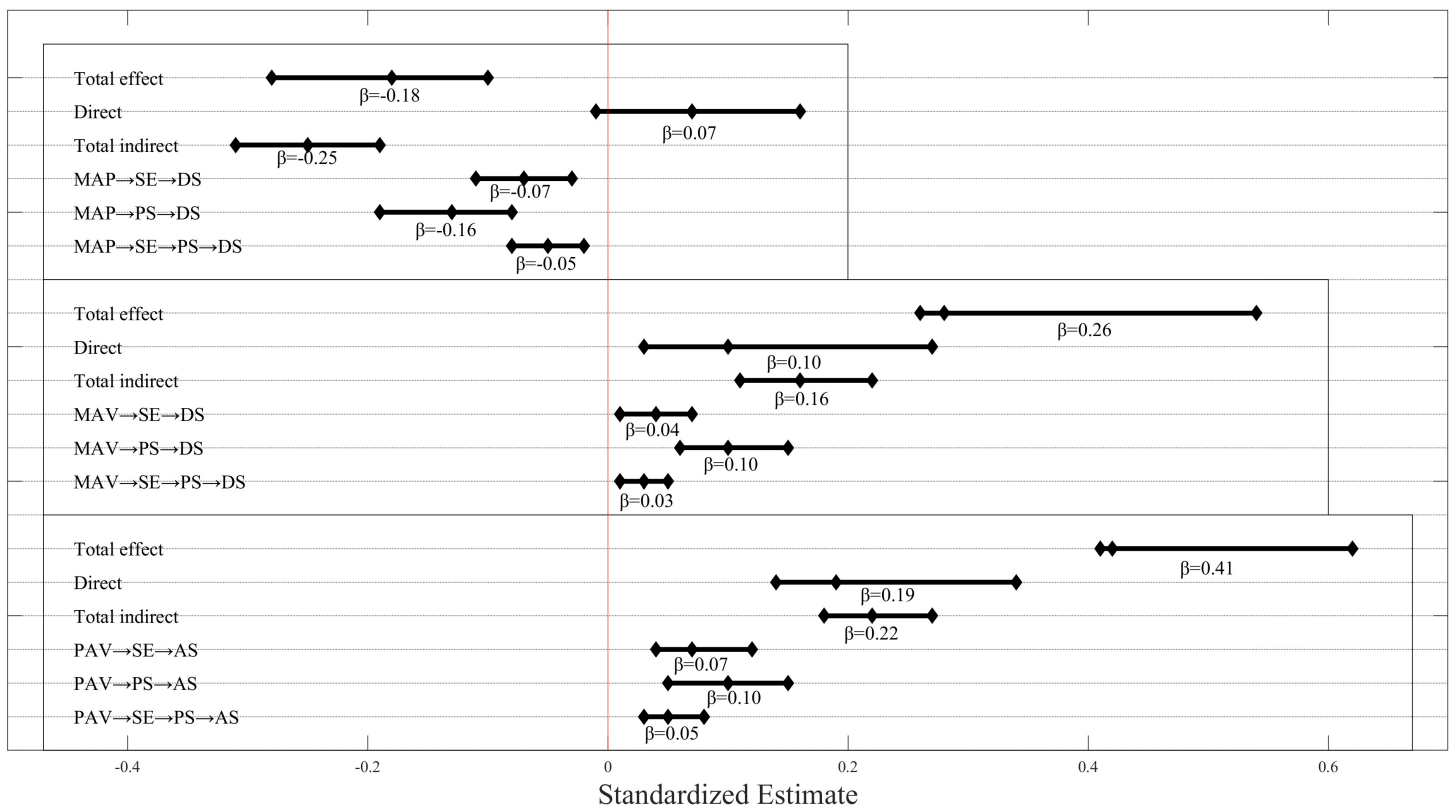
Effects from MAP, MAV, or PAV to AS.

Figure 3, the overall effect of MAP on AS was significant, with a negative prediction of anxiety (*β* = −0.18, 95% CI = [−0.28, −0.10]), a non-significant direct effect (*β* = 0.07, 95% CI = [−0.01, 0.16]), and a significant indirect effect (*β* = −0.25, 95% CI = [−0.31, −0.19]). The indirect path from MAP to AS was mediated by SE (*β* = −0.07, 95% CI = [−0.11, −0.03]), PS (*β* = −0.13, 95% CI = [−0.19, −0.08]), and SE → PS (*β* = −0.05, 95% CI = [−0.08, −0.02]), respectively. Self-esteem and perceived stress play a complete mediating role between mastery-approach goal and anxiety.

The results provided support for Hypothesis 1a, 2a, 3a, and 4a.

### Analysis of the effect of mastery-avoidance goal (MAV) on depression/anxiety

Table 3, MAV negatively predicted SE (*β* = −0.16, *p* < 0.001) and positively predicted PS (*β* = 0.28, *p* < 0.001). SE negatively predicted PS (*β* = −0.45, *p* < 0.001) and negatively predicted DS (*β* = −0.33, *p* < 0.001) and AS (*β* = −0.23, *p* < 0.001). PS positively predicted DS (*β* = 0.33, *p* < 0.001) and AS (*β* = 0.36, *p* < 0.001). Therefore, self-esteem and perceived stress mediated the relationship between mastery-avoidance goals and depression/anxiety.

**Table 3:** The direct and indirect effects of MAV on depression and anxiety.

	Model 1			Model 2			Model 3		
	Outcome: SE			Outcome: PS			Outcome: DS (AS)		
	SE	β	t	SE	β	t	SE	β	t
MAV	0.05	−0.16	−3.76^***^	0.06	0.28	7.61^***^	0.06 (0.06)	0.03 (0.10)	0.69 (2.50^*^)
SE				0.05	−0.45	−12.37^***^	0.06 (0.06)	−0.33 (−0.23)	−8.12^***^ (−5.58^***^)
PS							0.04 (0.04)	0.33 (0.36)	7.63^***^ (8.27^***^)
*R* ^2^	0.03			0.25			0.34 (0.31)		
*F*	14.11^***^			85.65^***^			88.68^***^ (78.93^***^)		

MAV, mastery-avoidance goal orientation; SE, self-esteem; PS, perceived stress; DS, depression; AS, anxiety. *p < 0.05, **p < 0.01, ***p < 0.001.

Figure 2, the overall effect of MAV on DS was significant and positively predicted depression (*β* = 0.20, 95% CI = [0.19, 0.47]). The direct effect was not significant (*β* = 0.03, 95% CI = [−0.08, 0.17]), while the indirect effect was significant (*β* = 0.17, 95% CI = [0.11, 0.23]). The indirect pathway from MAV to DS was mediated by SE (*β* = 0.05, 95% CI = [0.02, 0.10]), PS (*β* = 0.09, 95% CI = [0.05, 0.14]), and SE → PS (*β* = 0.02, 95% CI = [0.01, 0.04]), accounting for 25.00, 45.00, and 10.00% of the total effect, respectively. Self-esteem and perceived stress played a complete mediating role between mastery-avoidance goals and depression.

Figure 3, MAV had a significant overall effect on AS, positively predicting anxiety (*β* = 0.26, 95% CI = [0.28, 0.54]). The direct effect and indirect effect were significant (*β* = 0.10, 95% CI = [0.03, 0.27]; *β* = 0.16, 95% CI = [0.11, 0.22]). There were three indirect paths from MAV to AS, namely MAV → SE → AS (*β* = 0.04, 95% CI = [0.01, 0.07]), MAV → PS → AS (*β* = 0.10, 95% CI = [0.06, 0.15]), MAV → SE → PS → AS (*β* = 0.03, 95% CI = [0.01, 0.05]). The effect sizes of the three paths were 15.38, 38.46, and 11.54%. Self-esteem and perceived stress partially mediated the relationship between mastery-avoidance goals and anxiety.

These results supported Hypothesis 1b, 2b, 3b, and 4b.

### Analysis of the effect of performance-avoidance goal (PAV) on depression/anxiety

Table 4, PAV negatively predicted SE (*β* = −0.39, *p* < 0.001), while positively predicting PS (*β* = 0.29, *p* < 0.001). SE significantly and negatively predicted PS (*β* = −0.38, *p* < 0.001), DS (*β* = −0.30, *p* < 0.001), and AS (*β* = −0.19, *p* < 0.001). PS positively predicted DS (*β* = 0.29, *p* < 0.001) and AS (*β* = 0.33, *p* < 0.001). Self-esteem and perceived stress thus played a mediating role in the association between performance-avoidance goals and depression/anxiety.

**Table 4:** The direct and indirect effects of PAV on depression and anxiety.

	Model 1			Model 2			Model 3		
	Outcome: SE			Outcome: PS			Outcome: DS (AS)		
	SE	*β*	*t*	SE	*β*	*t*	SE	*β*	*t*
PAV	0.04	−0.39	−9.58^***^	0.06	0.29	7.45^***^	0.05 (0.05)	0.15 (0.19)	3.73^***^ (4.75^***^)
SE				0.06	−0.38	−9.83^***^	0.06 (0.06)	−0.30 (−0.19)	−7.22^***^ (−4.48^***^)
PS							0.04 (0.04)	0.29 (0.33)	6.81^***^ (7.74^***^)
*R* ^2^	0.15			0.32			0.35 (0.33)		
*F*	91.71^***^			122.36^***^			95.44^***^ (86.72^***^)		

PAV, performance-avoidance goal orientation; SE, self-esteem; PS, perceived stress; DS, depression; AS, anxiety. *p < 0.05, **p < 0.01, ***p < 0.001.

Figure 2, the overall effect of PAV on DS was significant, with a positive prediction of depression (*β* = 0.39, 95% CI = [0.42, 0.64]). There was also a significant direct effect (*β* = 0.15, 95% CI = [0.10, 0.31]) and a significant indirect effect (*β* = 0.24, 95% CI = [0.19, 0.30]). The indirect pathway from PAV to DS was influenced by the simple mediation of SE (*β* = 0.12, 95% CI = [0.07, 0.17]), the simple mediation of PS (*β* = 0.08, 95% CI = [0.04, 0.14]), and the serial mediation of SE to PS (*β* = 0.04, 95% CI = [0.02, 0.07]). The three mediating pathways contributed 30.77, 20.51, and 10.26% to the total effect. Self-esteem and perceived stress partially mediated the relationship between performance-avoidance goals and depression.

Figure 3, PAV had a significant overall effect on AS, positively predicting AS (*β* = 0.41, 95% CI = [0.42, 0.62]). The direct effect (*β* = 0.19, 95% CI = [0.14, 0.34]) and indirect effect (*β* = 0.22, 95% CI = [0.18, 0.27]) were also significant. There were three indirect pathways from PAV to AS, specifically, PAV → SE → AS (*β* = 0.07, 95% CI = [0.04, 0.12]), PAV → PS → AS (*β* = 0.10, 95% CI = [0.05, 0.15]), and PPAV→SE → PS → AS (*β* = 0.05, 95% CI = [0.03, 0.08]), explaining 17.07, 24.39, and 12.20% of the variance.

In summary, the above results validated Hypothesis 1c, 2c, 3c, and 4c.

## Discussion

This study discussed the relationship and internal mechanisms between achievement motivation and mental health. In particular, MAP negatively predicted depression/anxiety. MAV and PAV positively predicted depression/anxiety. Self-esteem played a mediating role in the relationship between achievement motivation and depression/anxiety. Perceived stress mediated the relationship between achievement motivation and depression/anxiety. Self-esteem and perceived stress played a chain intermediary role in the relationship between achievement motivation and depression/anxiety. There was no significant correlation between performance-approach goal (PAP) and depression/anxiety.

### Achievement goal and depression/anxiety

Descriptive statistical data showed achievement goals and overall mental health status of medical postgraduates. Research has shown that achievement motivation is dynamic, which exhibits diversity over different populations and time (Liu et al., 2023; Liu et al., 2024). Compared with other major postgraduates in China, there was no significant difference in the scores of medical postgraduates in MAP and MAV in this study. However, in terms of PAP (*t* = −3.757, *p* < 0.001) and PAV (*t* = −4.125, *p* < 0.001), the score of medical students was significantly lower than that of postgraduate students in other majors (Jia, 2017). There was no significant difference in the scores of mastery goals between the two groups, which may be because postgraduates in different majors need to acquire a lot of knowledge and improve their abilities during their postgraduate studies to meet academic requirements. However, the performance goals of medical students were lower than those of other professional postgraduate students, indicating that medical students do not overly care about positive or negative feedback from others when completing relevant learning tasks. Like other major postgraduates, medical master graduates not only require completion of research tasks, but also additional clinical work required for residency training (Xiong et al., 2024). Due to time constraints and heavy academic tasks, medical students are more concerned with whether tasks are completed on time, rather than their own academic rankings or evaluations from others. In addition, considering the dynamic nature of achievement motivation, we compared the achievement goals of medical postgraduate students with those of undergraduate medical students in China in this study (Wang et al., 2021). The results showed that compared with undergraduate medical students, the MAP (*t* = −4.538, *p* < 0.001), PAP (*t* = −12.232, *p* < 0.001), and PAV (*t* = −15.676, *p* < 0.001) of medical master’s students were significantly lower, while the MAV was significantly higher than that of medical undergraduate students (*t* = 9.459, *p* < 0.001). Undergraduates and postgraduate students differ in course content, learning methods, and training objectives. Undergraduate courses focus on comprehensive learning of basic medical knowledge and skills, while postgraduate studies place greater emphasis on depth and specialization. In contrast, undergraduate students have more enough study time and a stronger motivation to learn in order to master medical knowledge. However, medical postgraduates usually involves choosing a specific direction or entering a department for in-depth study, with a stronger purpose, direction, and autonomy to meet the requirements of clinical and research tasks. Therefore, the medical postgraduates tends to have lower MAP goals, while MAV goals are higher. Due to different training objectives, undergraduate students are more focused on their academic performance, while postgraduates are more concerned about publishing papers, and their attention to ranking and grades has decreased. This also determines that the PAP and PAV of medical postgraduates are frequently relatively low.

In this study, 22.45% of medical postgraduates reported moderate or severe depression, and 31.13% reported moderate or severe anxiety. These data were consistent with previous research findings, which suggest that approximately 20–30% of medical students experience depression or anxiety problems (Li et al., 2024; Wu and Ke, 2023; Zhao et al., 2019). Negative emotions not only pose a threat to the physical and mental health of medical students, but may also trigger a series of chain reactions. For example, Negative affect may lead to insomnia (Li et al., 2024), which in turn affects daily learning and life. In addition, negative emotions may also weaken the confidence and motivation of medical students, thereby having varying degrees of negative impact on their professional identity and future career performance (Duarte et al., 2022). More seriously, long-term negative emotions may even undermine medical students’ sense of professional mission, causing them to doubt and waver in their career choices (Wu and Ke, 2023). Medical postgraduate students are in a critical transition period from campus to society. They not only shoulder the dual roles of students and doctors, but also face severe challenges in clinical practice and increasingly fierce employment competition. In such a context, they may inevitably feel confused and anxious, but it is also an indispensable part of their growth process. Therefore, we must attach great importance to the mental health status of medical students and take effective measures to help them cope with negative emotions, in order to promote their comprehensive development and healthy growth.

Additionally, consistent with our hypotheses, this study found that MAP negatively predicted depression and anxiety (Hypothesis 1a), while MAV and PAV positively predicted depression/anxiety (Hypothesis 1b and 1c). These results validated previous research (Skaalvik, 2018; Steare et al., 2023). First, MAP was negatively correlated with depression/anxiety (Hypothesis 1b and 1c). Individuals with MAP hold positive achievement beliefs, characterized by deep learning (Phanudulkitti et al., 2018), and have less action crises when encountering difficulties (Katz-Vago and Benita, 2023). They view difficulties as challenges, with less pressure and emotional exhaustion (Tuominen-Soini et al., 2008). They also actively seek social support and feedback to alleviate psychological symptoms (Wang et al., 2021) and report higher happiness (Guo et al., 2023). Secondly, MAV was positively correlated with depression/anxiety (Hypothesis 1b). Approach goals are related to positive affect and behavioral activation systems, while avoidance goals are associated with negative affect and behavioral inhibition systems (Elliot and Thrash, 2002). Medical students who hold MAV pursue the growth of their abilities and make more efforts in studies. However, they worry that they cannot achieve their goals, thus facing more pressure, negative emotions, and cognitive anxiety, making them fear failure (Sideridis, 2008). Thirdly, PAV deepened depression/anxiety (Hypothesis 1c). Depression and anxiety are positively correlated with PAV (Sideridis, 2007), stress, and self-regulation failure (Sideridis, 2018). Another study found that insecure striving, shame, and self-compassion mediate between PAV and test anxiety (Santos, 2020). Individuals with PAV tend to worry about negative evaluation and poorer performance than others. To protect self-esteem, individuals may imply to themselves, “I am not incapable, I just have not put in enough effort.” Subsequently, procrastination frequently occurs among individuals with PAV (An and Jia, 2018; Chen, 2023), exacerbating their levels of depression and anxiety (Jiang, 2021; Zhang, 2023).

Thus, it can be observed that different achievement goals held by individuals can lead to varied emotional outcomes.

### The mediating role of self-esteem

This study found that self-esteem mediated the relationship between achievement goals and depression/anxiety, supporting hypotheses 2a, 2b, and 2c. For medical postgraduates, self-esteem negatively predicted psychological symptoms (Macinnes, 2006). Students with low self-esteem are more likely to be depressed or anxious (Greenberg et al., 1992; Orth et al., 2009a; Orth et al., 2009b; Rossi et al., 2020), with weaker motivation to recover from negative emotions after failures (Heimpel et al., 2002), resulting in poorer performance (Baumeister and Tice, 1985).

Besides, motivation affects psychological symptoms through self-esteem. Individuals with high self-esteem are driven by self-enhancement motivation, whereas those with low self-esteem are motivated by self-protection (Baumeister et al., 1989). The self-improvement motivation aligns with approach motivation, self-protection is similar to avoidance motivation (Heimpel et al., 2006). Therefore, high self-esteem is associated with approach motivation, while low self-esteem is related to avoidance motivation. The results also confirmed this. MAP and self-esteem alleviate psychological symptoms (hypothesis 2a). Students who hold MAP have higher self-esteem and do not care about external evaluations. They are unlikely to adopt self-protection and self-handicapping strategies (Schwinger and Stiensmeier-Pelster, 2011). Even if they fail, they will perceive it as a lack of effort (a controllable and modifiable factor) rather than an inability, making them far from emotional disorders. MAV was negatively correlated with self-esteem and mental illness (hypothesis 2b) (Liu, 2012). Individuals with MAV are dedicated to completing tasks accurately and without errors, driven by a fear of failure and concerns about their abilities to handle the current task. Consequently, their self-esteem is lower, leading to elevated depression and anxiety. The protective effect of self-esteem on depression/anxiety was influenced by PAV (hypothesis 2c). Individuals holding PAV may have lower self-esteem (Kandemir, 2014; Shim et al., 2012). Avoidance goals are related to a high behavioral inhibition system and low behavioral activation system (Elliot and Thrash, 2002), as well as fear of failure (Elliot and Sheldon, 1997) and insecure attachment (Elliot and Reis, 2003). For medical students driven by PAV, the fear of poor performance and denial about their abilities further lower their self-esteem. They may behave more negatively and passively when completing tasks, and thus experience deeper frustration and self-involvement (Liu et al., 2006), increasing their vulnerability to depression and anxiety.

Mental Health Education should be more targeted. High self-esteem medical students should be guided to use their internal psychological resources to cope with stress (Zhong, 2007). For students with low self-esteem, it is necessary to improve their learning motivation and engage in group counseling to enhance self-acceptance and self-esteem.

### The mediating role of perceived stress

This study replicated and extended previous research findings (Chi, 2018; Wang et al., 2021). Perceived stress mediated the relationship between achievement goals and depression/anxiety, confirming hypotheses 3a, 3b, and 3c. Perceived stress is positively correlated with negative emotions (Anyan and Hjemdal, 2016; Liu et al., 2021; Luo et al., 2021; Yang et al., 2021; Zhu L. et al., 2023). According to statistics, nearly 70% of primary depression is caused by stress, and stress accounts for 20–50% of the onset of depression (Hammen, 2005; Monroe and Harkness, 2005). Moreover, when individuals perceive excessive pressure and are unable to utilize existing resources to cope with the current situation, they may feel overwhelmed and anxious (Arpaia and Andersen, 2019). The relationship between stress and mental illness is a bidirectional prediction (Grant et al., 2004). Medical students face various pressures, which increase the risk of depression and anxiety (Tan, 2022; Wang et al., 2013; Ye et al., 2018).

Additionally, the study also found that achievement goals affect depression and anxiety through the mediating role of perceived stress. MAP negatively predicted perceived stress and depression/anxiety (Hypothesis 3a). Students who hold MAP perceive less pressure, prioritizing pursuing more challenges and long-term value goals (Chen, 2023). They view challenging tasks as opportunities, putting in more effort to achieve goals, and experiencing less negative emotions (Liu et al., 2006). MAV positively predicted perceived stress and depression/anxiety (Hypothesis 3b). Unlike medical students who study to develop their abilities, students holding MAV strive to show their capacities (Wang and Liu, 2023). Given the stringent academic and clinical standards expected of medical postgraduates, they need to split limited time to avoid clinical mistakes caused by inaccurate understandings of knowledge. These pressures exacerbate mental symptoms. PAV deepened depression/anxiety through the mediating effect of perceived stress (Hypothesis 3c). Students with high scores of PAV lack enthusiasm and initiative when facing learning and work, exhibiting low self-efficacy (Wang, 2012) and perceiving more pressure. Their engagement in learning is relatively low (Wang and Liu, 2023). This further leads to a decrease in their learning efficiency, and academic procrastination (Chen, 2023), exacerbating depression and anxiety.

### The series mediating effects of self-esteem and perceived stress

According to our hypotheses, self-esteem is related to perceived stress. Thus, self-esteem and perceived stress sequentially mediated the relationship between achievement goals and depression/anxiety (Hypotheses 4a, 4b, 4c). Threatening situations refer to the scenario perceived by individuals that may cause physiological or psychological harm or loss to themselves (Chi, 2018). Medical postgraduates face daily and challenging situations in clinical work and research activities. Failure to meet the required standards may bring pressure to students, undermining their self-esteem and status within the group. Self-esteem can influence individuals’ strategies for coping with threats and buffer the negative impact of stress or threats on medical students (Gerard and Buehler, 2004). Thus, perceived stress negatively predicts self-esteem.

### Performance-approach goal (PAP) and depression/anxiety

Of note, there was no significant correlation between PAP and depression/anxiety. Indeed, the impact of PAP is complex (Darnon et al., 2007), with contradictory research findings (Huang, 2011). Some researchers have proposed that the underlying causes of PAP deserve attention (Li et al., 2023). PAP characterized by strong autonomy is negatively correlated with anxiety and positively correlated with self-efficacy, whereas those characterized by strong control show positive correlations with anxiety and negative correlations with self-efficacy (Li et al., 2023). The linear correlation between PAP and depression/anxiety was not found in the present study, suggesting a complex relationship between both variables (Wang et al., 2021). Further attention may be needed on the role of other motivational variables.

## Implications and limitations

Previous studies have seldom investigated the internal mechanisms of achievement motivation and mental health. The current study extended prior research by integrating the personality trait of self-esteem with the environmental factor of perceived stress for the first time. We have put forward some research suggestions based on the research conclusions. Firstly, future research should also consider the role of other variables in the relationship between achievement motivation and depression anxiety, such as individuals’ coping styles with stress and their level of engagement in academics. Secondly, due to the dynamic changes in achievement motivation and mental health, it is particularly important to conduct research on the longitudinal trajectory of postgraduate students’ achievement motivation and depression/anxiety. Furthermore, most of the current research on achievement motivation is quantitative, and future research can use qualitative methods such as text analysis, single-case research design, and interview to explore the relationship between achievement motivation and psychological status. For example, in the current context of artificial intelligence, future researchers may use technologies such as API interfaces, web crawlers, and natural language processing (NLP) to collect and crawl comments, posts, and related research content from graduate students on social media, forums, blogs, and other platforms. Researchers could perform feature extraction and sentiment analysis, construct machine learning models, and visualize the data to analyze the psychological status and achievement motivation of the postgraduates, thereby supplementing existing quantitative research results. Lastly, researchers and practitioners in the field of psychology should collaborate to design and develop online and offline group counseling activities with achievement motivation as the main theme. They should also design virtual simulation experiments based on online group counseling projects to achieve the goal of practicing and growing anytime, anywhere. Researchers should design experiments to observe and measure the improvement effects of group counseling activities on students’ achievement motivation, learning engagement, and psychological state.

Moreover, the current study holds significant practical value. Firstly, attention should be paid to the pressures faced by medical students. Regular lectures or group counseling should be held to improve students’ cognition and coping strategies related to stress. Secondly, counselors should emphasize the role of achievement motivation, reduce students’ performance-avoidance and mastery-avoidance goals through lectures and training, and promote mastery-approach goals. Thirdly, universities and hospitals should concentrate on developing practical scientific research and clinical skills among medical students, thereby boosting their confidence in handling academic challenges. Finally, based on the different needs and characteristics of medical students, diverse motivational methods should be adopted to stimulate their competitiveness and initiative.

This study also had certain limitations. Methodologically, a cross-sectional design was adopted, limiting the inference of causal relationships between variables. Longitudinal studies could be employed to further investigate the longitudinal trajectory of achievement motivation among medical postgraduate students. In addition, the medical students from Shanxi Province were recruited. Future research should include medical postgraduates from diverse regions of China to expand sample representativeness. Finally, the utilization of self-report scales involved subjectivity and social desirability effect.

## Data Availability

The raw data supporting the conclusions of this article will be made available by the authors, without undue reservation.
