# Correction: Achievement motivation and mental health among medical postgraduates: the chain mediating effect of self-esteem and perceived stress

**DOI:** 10.3389/fpsyg.2025.1712745

**Published:** 2025-10-15

**Authors:** Mu-yun Ma, Yao Li, Li Guo, Guan-e Yang

**Affiliations:** ^1^Department of Applied Psychology, School of Humanities and Social Sciences, Shanxi Medical University, Jinzhong, China; ^2^Department of Traditional Chinese Pharmacology, School of Pharmaceutical Sciences, Shanxi Medical University, Jinzhong, China

**Keywords:** medical postgraduates, achievement motivation, depression, anxiety, self-esteem, perceived stress

A portion of text was captured incorrectly in the **Abstract and Results section**. The original text appears below:

“there was no significant linear correlation between mastery-approach goals and depression/anxiety”.

This corrected text appears below:

“there was no significant linear correlation between performance-approach goals and depression/anxiety”.

The original version of this article has been updated.

